# Targeted Glucose or Glutamine Metabolic Therapy Combined With PD-1/PD-L1 Checkpoint Blockade Immunotherapy for the Treatment of Tumors - Mechanisms and Strategies

**DOI:** 10.3389/fonc.2021.697894

**Published:** 2021-07-13

**Authors:** Guofeng Ma, Chun Li, Zhilei Zhang, Ye Liang, Zhijuan Liang, Yuanbin Chen, Liping Wang, Dan Li, Manqin Zeng, Wenhong Shan, Haitao Niu

**Affiliations:** ^1^ Department of Urology, The Affiliated Hospital of Qingdao University, Qingdao, China; ^2^ Key Laboratory, Department of Urology and Andrology, The Affiliated Hospital of Qingdao University, Qingdao, China; ^3^ Department of Pharmacy, Central Hospital of Shengli Oil Field, Dongying, China; ^4^ Department of Pathology, The Affiliated Hospital of Qingdao University, Qingdao, China; ^5^ Department of Nephrology, Qingdao Central Hospital, The Second Clinical Medical College of Qingdao University, Qingdao, China

**Keywords:** immunotherapy, PD-1/PD-L1 immune checkpoint, glucose metabolism, glutamine metabolism, combination therapy, tumor microenvironment

## Abstract

Immunotherapy, especially PD-1/PD-L1 checkpoint blockade immunotherapy, has led tumor therapy into a new era. However, the vast majority of patients do not benefit from immunotherapy. One possible reason for this lack of response is that the association between tumors, immune cells and metabolic reprogramming in the tumor microenvironment affect tumor immune escape. Generally, the limited amount of metabolites in the tumor microenvironment leads to nutritional competition between tumors and immune cells. Metabolism regulates tumor cell expression of PD-L1, and the PD-1/PD-L1 immune checkpoint regulates the metabolism of tumor and T cells, which suggests that targeted tumor metabolism may have a synergistic therapeutic effect together with immunotherapy. However, the targeting of different metabolic pathways in different tumors may have different effects on tumor immune escape. Herein, we discuss the influence of glucose metabolism and glutamine metabolism on tumor immune escape and describe the theoretical basis for strategies targeting glucose or glutamine metabolism in combination with PD-1/PD-L1 checkpoint blockade immunotherapy.

## Introduction

The traditional method of cancer treatment is surgical removal, followed by two different types of treatment: radiotherapy plus chemotherapy and targeted therapy that inhibit tumor angiogenesis and oncogenic signaling. However, these therapies are usually effective for only early-stage cancers and usually cannot cure advanced-stage cancers and have substantial side effects. Research on resistance to radiotherapy and chemotherapy usually focuses on tumor cell-intrinsic effects, such as cell cycle arrest and cell death caused by DNA damage, the switching of survival signalling pathways and the activation of new signaling pathways that lead to tumor resistance (1, 2). Research on resistance to targeted drugs mainly focuses on gene mutations. Gene mutations usually occur after the administration of targeted agents, such that the targeted drugs cannot bind to the target tumor molecules. The reprogramming of biological systems is also a reason for drug resistance (3). Therefore, methods to improve treatment mainly focus on the activation of new signaling pathways and the inherent genetic heterogeneity of tumor cells. However, these methods still cannot solve the problem of tumor drug resistance. The present study further examines tumor immunotherapy and metabolism in the tumor microenvironment (TME) in order to find a better way to treat tumors.

The study of the cancer-immunity cycle led to the development of checkpoint immunotherapy (4, 5). The PD-1/PD-L1 pathway is one of the core pathways involved in tumor immune escape, and it received the most attention in recent years. PD-L1 and PD-1 are important inhibitory costimulatory molecules that regulate the immune response of T cells, and these cells are also known as immune checkpoint molecules (6). PD-1 is expressed on activated T cells, and its ligands PD-L1 and PD-L2 are expressed on immune cells and tumors. PD-1 binds to PD-L1 on tumor cells and drives T cells into a dysfunctional state (7). Pembrolizumab and Nivolumab are two antibodies that inhibit PD-1 interactions with its ligands, and these agents are approved by the FDA for the treatment of non-small cell lung cancer, advanced melanoma, and renal cell carcinoma (8, 9). Although the development of PD-1/PD-L1 checkpoint immunotherapy has greatly promoted the progress of tumor treatment strategies, most patients do not respond to treatment or have off-target effects in clinical trials. These shortcomings may be attributed to genetic mutations (10), the status of immune infiltration in the tumor (11) and the expression of PD-L1 by tumors and immune cells (12, 13). Therefore, it is necessary to design a more reasonable combination therapy strategy to improve the effect of checkpoint immunotherapy.

Metabolic reprogramming is a hallmark of malignant tumors. Different stages of tumor progression are accompanied with different types of metabolic reprogramming. These different metabolic phenotypes may provide strategies for the targeted metabolic treatment of tumors (14). The Warburg effect is a typical example of metabolic reprogramming that is controlled by oncogenes. Under aerobic conditions, tumor cells still rely on the conversion of glucose to lactic acid for energy, which provides sufficient energy for tumor proliferation and produces a specifically acidic TME that can inhibit the function of T cells, and it is more conducive to tumor progression (15, 16). Glutamine is another important nutrient involved in tumor progression that can regulate tumor energy production, signal transduction and redox homeostasis. Glutamine transporter variants such as SLC1A5 have been shown to promote tumor metabolic reprogramming (17). The main problem facing tumor metabolism treatment strategies is how to specifically target tumor metabolism without affecting normal cell metabolism and inhibiting the function of antitumor immune effect cells in the TME. In addition, owing to the diversity of cell metabolism pathways, there are numerous metabolic bypass pathways, and the compensation of these pathways also limits the effect of targeting metabolism. Therefore, it is necessary to further study tumor metabolism pathways to understand the relationship between tumor metabolism and tumor immunity in the TME, and design more reasonable combination immunotherapies and targeted metabolic therapy strategies to treat tumors more effectively.

In this review, we discuss two strategies targeting tumor glucose metabolism or glutamine metabolism and explore how the targeting of glucose or glutamine metabolism affects tumor immune escape *via* the regulation of tumor PD-L1 expression and the function of T cells in addition to their direct effects on the tumor’s own biological activity. In this way, we further reveal the mechanism underlying strategies that combine targeted glucose or glutamine metabolic with PD-1/PD-L1 checkpoint blockade immunotherapy and provide a strong rationale for these strategies in the treatment of tumors.

## Mechanism of Glucose or Glutamine Metabolism-Targeting Therapy and PD-1/PD-L1 Checkpoint Blockade Immunotherapy Combinations for the Treatment of Tumors

### Tumor Glucose Metabolism and Immune Escape

The difference in metabolism between tumor and normal tissues suggests a reprogramming of tumor metabolism. Unlike normal cells, tumor cells can use large amounts of glucose to produce lactic acid through glycolysis in the cytoplasm even in the presence of high oxygen. In addition, this kind of high glycolytic flux produces large amounts of ATP, with a correspondingly low rate of oxidative phosphorylation (OXPHOS) in mitochondria. This phenomenon is called the Warburg effect (15, 16, 18). The further study of tumor glucose metabolism revealed that tumors often represent a mosaic of tumor cells with different metabolic characteristics. Some tumors rely more on oxygen, and other tumors are more prone to glycolysis (19). For example, metabolic heterogeneity of glucose metabolism exists within and between human lung tumors, human clear cell renal cell carcinomas (ccRCCs), high-grade serous ovarian and pancreatic cancer (20–23). However, the Warburg effect is not only important for energy purposes but also to provide building blocks for synthesis of macromolecules for tumors that rely on this effect, and it may be used as a marker of tumor malignancy (24–28). Traditionally, the Warburg effect enables tumors to obtain the large amount of energy that is needed for rapid proliferation, which promotes tumor growth and metastasis (29, 30). Recent studies have shown that the Warburg effect also played an important role in the tumor immune escape mechanism (31–33).

#### Glycolysis Regulates the Expression of PD-L1 in Tumor Cells

**Figure 1:**
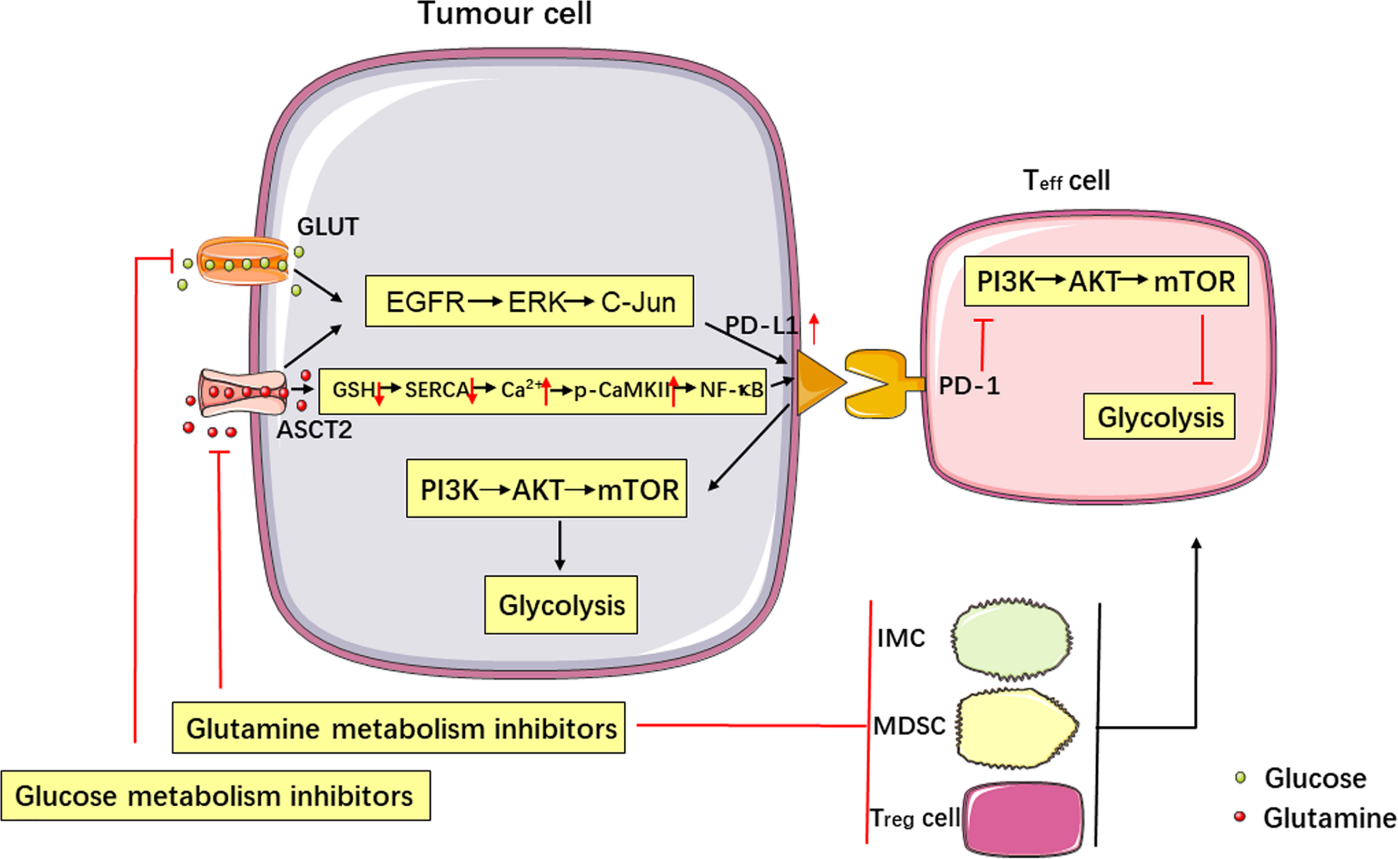
Metabolism affects tumor immune escape, and the PD-1/PD-L1 immune checkpoint regulates metabolic pathways. Glucose and glutamine metabolism upregulate the expression of PD-L1 in tumor cells *via the* EGFR/ERK/C-Jun pathway. Inhibition of glutamine use in tumor cells increases PD-L1 expression by reducing the levels of GSH, inhibiting the SERCA activation, and increasing cytosolic Ca2+ levels and CaMKII phosphorylation, which further activates the downstream NF-κB signalling pathway. Targeting glutamine metabolism can inhibit the production of immune cells negatively affecting the immune response (IMCs, MDSCs and T_reg_ cells) and upregulate the function of T_eff_ cells, thereby enhancing the antitumor immune response. Activation of the PD-L1/PD-1 signalling pathway promotes aerobic glycolysis (i.e., the Warburg effect) in tumor cells, inhibits glucose metabolism in T_eff_ cells by stimulating the PI3K-AKT-mTOR signalling pathway, and produces synergistic inhibition of the antitumor response.

The Warburg effect is the main energy source of some tumors, and this reliance causes tumor cells to consume a large amount of glucose to survive. Imperfect blood vessel development in solid tumors leads to a limited supply of nutrients for tumors. Therefore, glucose in the TME is often lacking (33–35). PD-L1 is a negative immunoregulator that is regulated by glycolysis in many tumors. *In vitro* experiments found that a reduction in glucose content in the culture medium upregulated the expression of PD-L1 in renal cancer cells *via* the EGFR/ERK/C-Jun pathway (36). The expression of PD-L1 correlated with the uptake of ^18^F-FDG in lung adenocarcinoma (37). Pyruvate kinase is the key enzyme in the last step of glycolysis. The isoenzyme pyruvate kinase isozyme type M2 (PKM2) has been shown to promote the expression of PD-L1 in tumor and immune cells. The use of the PKM2 activator TEPP-46 to increase the conversion of phosphoenolpyruvate to pyruvate reduces the expression of PD-L1 in a murine CT26 colon carcinoma model and in tumors (38). TCGA database analysis also proved that glycolytic activity was related to active immune characteristics in various cancers, and *in vitro* experimental studies have proved that glycolysis can increase the expression of PD-L1 in breast cancer, osteosarcoma and ovarian cancer (39). Therefore, tumors with glucose-deficient TME regulate the expression of PD-L1 *via* glycolysis, which may cause tumor immune escape **(** **Figure 1** **)**.

#### The PD-1/PD-L1 Immune Checkpoint Regulates Glucose Metabolism in Tumors and T Cells

In recent years, PD-1/PD-L1 immune checkpoint blockade (ICB) therapy has made significant progress in the treatment of tumors. This type of therapy is based on the interaction between PD-L1 and PD-1, which inhibits the activation and proliferation of T cells and mediates tumor immune escape. Increasing evidence shows that the interaction of PD-L1 and PD-1 regulates the glucose metabolism of tumors and T cells, which affect the nutrient competition between these two cell types in the TME. The interaction of PD-1 with PD-L1 or PD-L2 on T cells impairs aerobic glycolysis *via* inhibition of the PI3K-AKT-mTOR pathway (40). The expression of PD-L1 on cancer cells drives activation of the PI3K-Akt-mTOR pathway to stimulate aerobic glycolysis, increases glucose uptake and enhance T cell competition for glucose (41–43). Therefore, the PD-1/PD-L1 axis can synergistically promote tumor immune escape *via* the upregulation of tumor cell glycolysis and inhibiting of T cell glycolysis **(****Figure 1****)**.

#### The Warburg Effect Forces T Cells to Use the Limited Supply of Glucose in the TME

**Figure 2:**
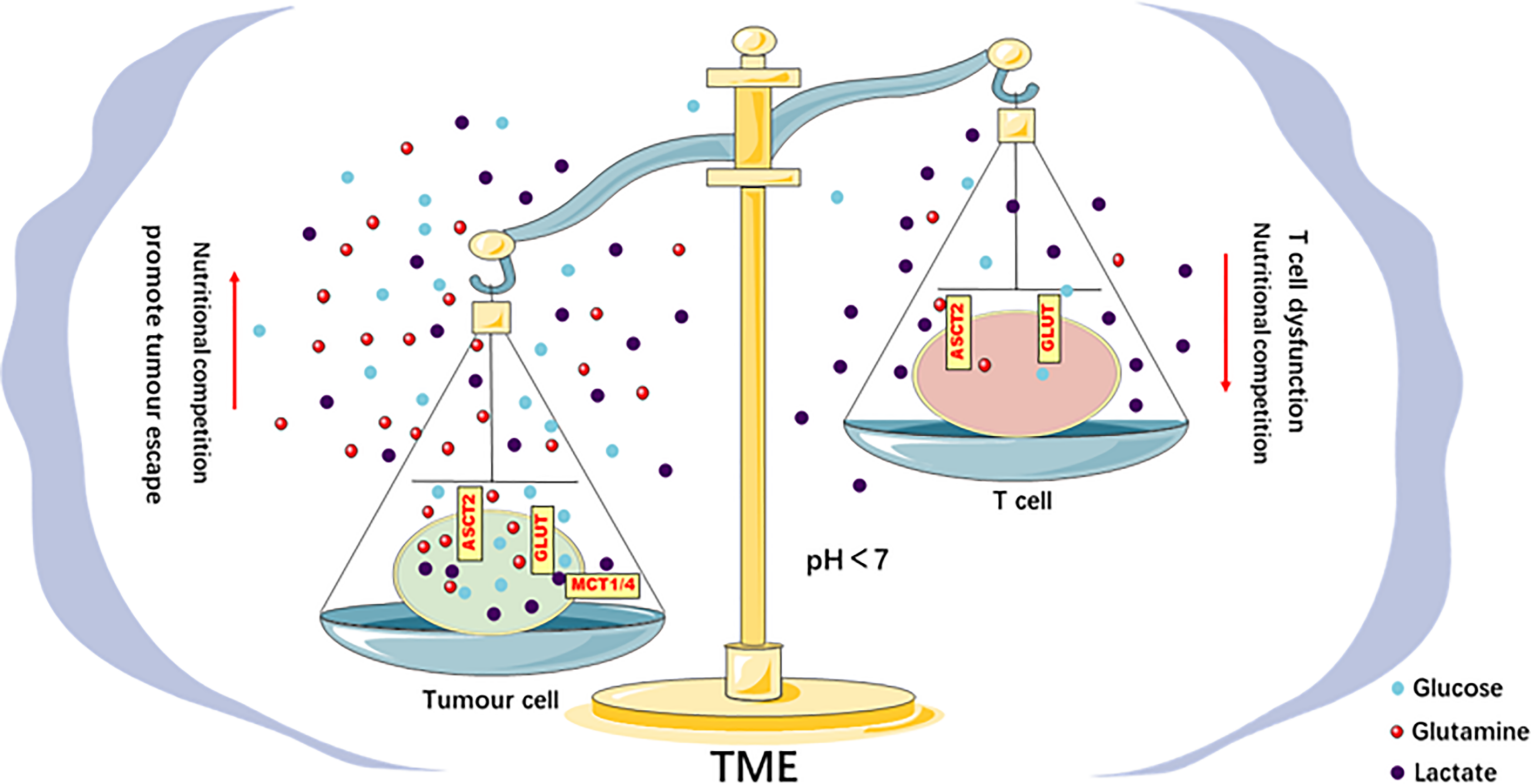
Metabolic competition in the TME drives tumor progression. There is competition for glucose and glutamine between tumor and T_eff_ cells in the TME. This competition leads to the limited use of energy materials by T_eff_ cells and impairs their function, which promotes immune escape. The acidic TME caused by the lactic acid produced by the tumor Warburg effect inhibits the function of T_eff_ cells, impairs the antitumor immune response, and promotes tumor progression.

The different glucose metabolism modes of tumors and T cells suggest competition between these types of cells for glucose use in the TME. The Warburg effect supports the rapid growth of tumors and the demand for macromolecules, which provides an external advantage for tumor cells, accelerates the consumption of glucose in the TME, and limits the glucose uptake of tumor-infiltrating T cells, which results in dysfunction (44, 45). For example, the effector function of CD4^+^ and CD8 ^+^ T_eff_ cells was reduced under low-glucose conditions, and the production of interferon-γ (IFN-γ), intermediate-17 (IL-17) and granzyme B was inhibited (46–48). The production of an intermediate of T cell glycolysis, phosphoenolpyruvate (PEP), was inhibited, which interfered with the signal transduction of the calcium-dependent transcription factor nuclear factor of activated T cells (NFAT) (42). These results indicate that glucose metabolism directly controls the activation of T cells, and the limited use of glucose by T cells in the TME damages their functions and reduce immune responses. Primary ovarian cancer cells in a coculture system suppressed the expression of the methyltransferase EZH2 by maintaining high expression of the microRNAs miR-101 and miR-26a and imposing glucose restriction on T cells, which inhibited T cell function (49). Increasing the glycolytic ability of mouse sarcoma cells led to the inhibition of CD8^+^ T cell function in a coculture system (41). The function of CD4^+^ T cells was inhibited in a mouse model of melanoma overexpressing HK2. Overexpression of the glycolytic enzyme PEP carboxykinase in tumor-specific CD4^+^ T cells enhanced the antitumor effect (42). These results indicate that the function of effector T cells in the TME is related to the glycolytic activity of tumors, and tumors cause T cell metabolism disorders *via* glucose competition and effects on tumor immune escape **(****Figure 2****)**.

#### The Acidic TME Induced by the Warburg Effect Inhibits the Function of T Cells

The Warburg effect of tumor cells produces a large amount of lactic acid. Lactic acid plays a different role in tumor cells than it does in T cells. Lactic acid establishes metabolic coupling between tumor and nonmalignant cells or between tumor cells to maintain tumor growth (50). Genetically modified mouse models of lung and pancreatic cancer found that circulating lactic acid was used to generate energy and induce the release of glucose to promote tumor growth (51). In contrast, the acidic TME formed by the large amount of lactic acid produced by tumor glycolysis inhibited the proliferation, survival, cytotoxicity and cytokine production of T cells (52, 53). *In vitro* experiments found that the activation process of mouse CD8^+^ T cells was dysfunctional under high-lactate and high-H^+^ conditions, and MAP kinase signal transduction during the activation of human effector CD8^+^ T cells was seriously damaged (53, 54). Under acidic pH conditions, PSGL-1 can act as a selective receptor for VISTA to inhibit the activity of T cells, which ultimately leads to immune escape of tumor cells (55). The low lactate levels produced by mouse melanoma cells in an LDHA knockout mouse melanoma model mediated a strong tumor exclusion response (53). These results indicate that the acidic TME mediated by the Warburg effect is beneficial to tumor progression but inhibits the function of T cells, which may cause tumor immune escape **(****Figure 2****)**.

In conclusion, tumor glucose metabolism can affect tumor immune escape *via* regulation of the expression of PD-L1 in tumors and its effects on the function of T cells in TME *via* different pathways. Therefore, the influence of tumor glucose metabolism on tumor immune escape should be considered for interventions.

## Tumor Glutamine Metabolism and Immune Escape

Glutamine is one of the most abundant amino acids in the human body, and it is an indispensable energy source for tumor survival and progression. Many tumor cells rely on glutamine for survival. Glutamine metabolism provides energy for tumor cells by producing ATP and participating in the tricarboxylic acid cycle. Glutamine also provides raw materials for the synthesis of macromolecular substances in tumor cells, such as nucleotides and hexosamine (56). Notably, glutamine metabolism is involved in tumor proliferation and metastasis (57, 58). Previous studies have demonstrated a correlation between tumor progression and glutamine metabolism. Tumor cells rely on glutamine metabolism for energy and the synthesis of macromolecules even when aerobic glycolysis provides sufficient energy (59). Glutamine metabolism provides high levels of NADPH for tumor cells to maintain the redox state and ensure the survival of tumor cells (60). Recent studies have shown that glutamine metabolism also affected tumor immune escape.

### Glutamine Metabolism Regulates the Expression of Tumor PD-L1 and Regulatory Immune Cells Activity

Glutamine metabolism maintains tumor proliferation and progression, and the targeting of glutamine metabolism can effectively inhibit tumor growth. Notably, recent studies have shown that targeted glutamine metabolism affected tumor immune escape *via* different mechanisms during tumor growth inhibition. Glutamine deprivation in the culture medium upregulated the expression of PD-L1 on renal cancer cells *via* the EGFR/ERK/C-Jun pathway (61). The inhibition of glutamine use in lung and colon tumors increased the expression of PD-L1 by reducing GSH levels. The reduction in GSH levels inhibited sarcoplasmic reticulum Ca^2+^-ATPase (SERCA) activation and increased cytosolic Ca^2+^ levels and CaMKII phosphorylation, which further activated the downstream nuclear factor-kappa B (NF-κB) signalling pathway to promote the expression of PD-L1 (62). However, researchers found that glutamine deprivation in renal cancer cells can weaken an immunosuppressive TME. Glutamine deprivation induced M2 macrophages to secrete IL-23 *via* activation of the hypoxia-inducible factor 1 alpha (HIF1α) pathway. IL-23 inhibits the proliferation and activation of T_reg_ cells, which upregulates the proliferation and activity of CD4^+^ and CD8^+^ T_eff_ cells, to enhance the antitumor immune response (63). The use of a small-molecule inhibitor of glutamine metabolism demonstrated that blockade of glutamine metabolism inhibited the generation and recruitment of myeloid-derived suppressor cells (MDSCs) *via* inhibition of the production of colony-stimulating factor 3 (CSF3), which enhanced the function of T cells in TME (64). Another study showed that glutamine metabolism can regulate the production of immature myeloid cells (IMCs) with a highly immunosuppressive effect. Blockade of glutamine metabolism improved the therapeutic effect of anti-PD-L1 agents in an ICB therapy-resistant mouse model, which indicates that glutamine metabolism can regulate the antitumor immune response (65). All of these data indicate the complexity of targeting glutamine metabolism to regulate the immune response because these strategies may simultaneously cause tumor immune escape *via* upregulation of the expression of PD-L1 in tumors and inhibition of tumor immune escape by enhancing the function of T cells **(****Figure 1****)**.

### Glutamine Competition Leads to Restriction of Glutamine Use by T Cells in the TME

Some tumor cells require a high consumption of glutamine, in addition to glucose, to meet tumor progression needs. Therefore, there is also competition for glutamine between tumors and T cells in the TME. As mentioned above, the glutamine metabolic pathway in tumor cells affects the proliferation and activation of T cells, and glutamine deprivation inhibits T cell proliferation and cytokine production (66, 67). Studies have shown that the MAPK/ERK pathway upregulates glutamine uptake by T cells in the process of T cell activation. The MAPK/ERK pathway also upregulates glutamine uptake and use by tumor cells (67, 68). Therefore, the MAPK/ERK pathway may play an important role in the competition for glutamine between T cells and tumor cells. Triple-negative breast cancer cells competitively deprive glutamine in the TME and limits the use of glutamine by tumor-infiltrating T cells, which affects the function of T cells and damages antitumor immune responses. The concentration of glutamine in the tumor interstitium was increased in a tumor model with specific GLS loss, and the activity of intratumoral T cells was increased. Limiting glutamine in the TME also differentially affected tumors and T cells. For example, the glutamine transporter inhibitor V-9302 selectively inhibits glutamine uptake by tumor, but not CD8^+^ T, cells. CD8^+^ T cells can regulate glutamine metabolism by upregulating glutamine transporter ATB^0,+^/Slc6a14 (69). The glutamine antagonist 6-diazo-5-oxo-L-norleucine (DON) and its precursor, JHU-083, inhibit tumor metabolism and have a strong antitumor effect. In contrast, the antitumor effects of CD8^+^T cells may be increased *via* metabolic reprogramming strategies that upregulate glycolysis and oxidative metabolism (70). These data show that tumor cells competitively use glutamine, which limits the use of glutamine by T cells and affects T cell function. The targeting of glutamine metabolism can inhibit tumor cell proliferation and relieves the restriction of glutamine use by tumor cells, which enhances the function of T cells **(****Figure 2****)**.

In summary, tumor glutamine metabolism can regulate the function of T cells and affects tumor immune escape by regulating the expression of tumor PD-L1, the activation of regulatory immune cells and nutritional competition. Therefore, it is necessary to fully consider the effects of the upregulation of tumor PD-L1 expression and enhancing the function of T cells on tumor immune escape when intervening in tumor glutamine metabolism.

## Strategies Targeting Glucose or Glutamine Metabolism In Combination With PD-1/PD-L1 Checkpoint Blockade Immunotherapy for Tumor Treatment

Immunotherapy produced fundamental changes to cancer treatment. Immune checkpoint inhibitors (ICIs), especially PD-1/PD-L1 ICIs, achieved amazing therapeutic effects in a variety of tumor types. However, there are a large number of people in whom ICIs are ineffective or who gradually develop drug resistance during the process of treatment. Therefore, solving the problem of drug resistance in immunotherapy has become a hot area of research. Previous studies attributed the failure of ICI therapy to different types of T cell production disorders or dysfunctions, such as insufficient antitumor T cell production, insufficient tumor-specific T cell function, and impaired T cell immune memory function (71). Increasing the generation and function of T_eff_ cells and reducing the generation and function of T_reg_ cells have become key goals for solving the problem of drug resistance in immunotherapy. As mentioned above, the different effects of glucose and glutamine metabolism on tumor and T cells determine the effect of metabolic interactions between tumor and T cells on tumor immune escape. Glucose metabolism and glutamine metabolism not only affect tumor proliferation and immune effects but also have different effects on different types of T cells. Therefore, strategies targeting glucose or glutamine metabolism in combination with PD-1/PD-L1 checkpoint blockade immunotherapy should enhance the power of cancer immunotherapy.

### Targeting Glucose Metabolism in Combination With PD-1/PD-L1 Checkpoint Blockade Immunotherapy for Tumor Treatment

**Figure 3:**
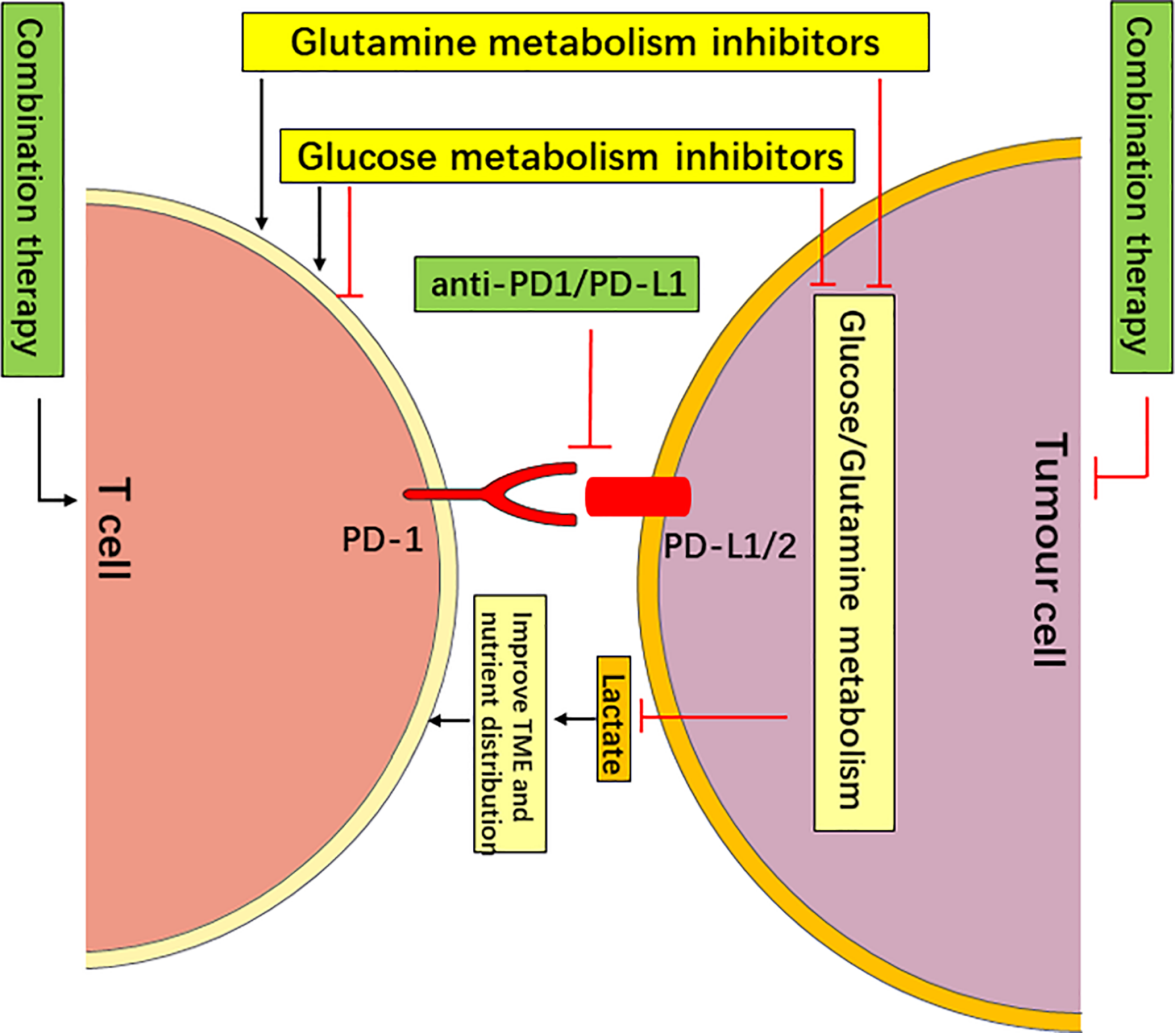
Targeting Metabolism in Combination with PD-1/PD-L1 Checkpoint Blockade Immunotherapy May Have a Synergistic Anticancer effect. The targeting of glucose or glutamine metabolism has an antitumor effect by starving tumors, and it improves the nutrient distribution and acidic TME, which is more conducive to the function of T cells. The targeting of glutamine metabolism may improve the function of T cells *via* metabolic reprogramming (such as DON and V-9302). However, the targeting of glucose metabolism may enhance or inhibit the function of different types of T cells (such as 2-DG). Therefore, the targeting of glucose or glutamine metabolism in combination with PD-1/PD-L1 checkpoint blockade immunotherapy may have a synergistic anti-cancer effect.

The Warburg effect provides an abundance of energy and intermediates for tumor metabolism to maintain tumor progression. Tumor cells also regulate the function of T cells *via* glucose competition, lactic acid acidification of the TME and direct regulation of PD-L1 expression. The PD-1/PD-L1 axis upregulates tumor cell glycolysis and simultaneously inhibits T cell glycolysis. Current strategies targeting glucose metabolism for the treatment of tumors mainly target rate-limiting enzymes of glycolysis, such as hexokinase 2 (HK2), PKM2, phosphofructokinase-2/fructose-2,6-bisphosphatase 3 (PFKFB3) and lactate dehydrogenase (LDHA) (38, 72–74). The relationship between tumor glucose metabolism and the tumor immune escape provides a theoretical basis for strategies targeting glucose metabolism in combination with PD-1/PD-L1 checkpoint blockade immunotherapy for tumor treatment. Therefore, targeted glucose metabolism in combination with PD-1/PD-L1 checkpoint blockade immunotherapy for tumor treatment will further improve the therapeutic effect. This is mainly based on the following ideas: ① The targeting of glucose metabolism may limit the use of glucose by tumors and upregulate glucose in the TME, which is more conducive to the function of T_eff_ cells. Therefore, the concurrent use of PD-1/PD-L1 ICBs may further improve the function of T cells. ② The targeting of glucose metabolism inhibits tumor cell production of lactic acid, improves the acidic TME, and weakens the inhibitory effect on the function of effector T cells. The combined use of PD-1/PD-L1 ICBs may further enhance the function of T cells. ③ The targeting of glucose metabolism may upregulate the expression of PD-L1 in tumor cells, and the simultaneous use of PD-L1 monoclonal antibody may improve the body’s antitumor immune response and produce synergistic anticancer effects. ④ The interaction of PD-1 and PD-L1 can not only inhibit the activation and proliferation of T cells but also upregulate the glycolysis of tumor cells and inhibits the glycolysis of T cells. The targeting of glucose metabolism in combination with PD-1/PD-L1 ICBs can relieve this effect and improve the body’s antitumor immune response **(****Figure 3****)**.

Although none of these combination therapies are approved for clinical treatment, the results of relevant experiments have proven the superiority of combination therapy. For example, metformin, which affects glucose metabolism and levels, reduced the hypoxic state of xenograft tumors, which rendered the tumors responsive to PD-1 blockade and enhancing the function of immune cells (75). PFKFB3 promoted the glycolysis of tumors and the production of lactic acid (76), and inhibition of PFKFB3 eliminated the Warburg effect, inhibit tumor progression and metastasis (77), and improved the therapeutic response to antibodies targeting the inhibitory immune checkpoint receptor in a mouse B16 melanoma model (78). LDHA is a key catalytic enzyme for aerobic glycolysis. Inhibition of LDHA reduced tumor growth in a xenograft model (79, 80), and researchers have proven that low levels of LDHA were associated with better anti-PD-1 antibody therapeutic responses in patients with melanoma (81). These experimental results support that the targeting of glycolysis enhances the effect of anti-PD-1/PD-L1 immunotherapy. Several current combinatory therapies are being tested in clinical trials **(** **Table 1** **)**. However, the current challenge is how to target the glucose metabolism of tumor cells without affecting the glucose metabolism of immune and normal cells. In fact, it has been proved that inhibiting of glycolytic enzymes or use of the competitive glucose analogue 2-DG can support the formation of long-term memory CD8+ T cells, but it also inhibits the proliferation and function of tumor-infiltrating effector immune cells (82–85).Current unnatural sugar metabolism labeling technology achieved a preferential labeling of cancer cells and targeted delivery (86). The combination of imaging technology and isotope-labeling technology provides a “visualization” solution for targeted tumor metabolism. However, the clinical application of these therapies requires more time, and more research is needed to solve these problems in the future.

**Table 1:** Currently ongoing trials of glucose and glutamine metabolic interventions combined with immune checkpoint inhibitors.

Metabolic agent	Immune-checkpoint inhibitor	Cancer types	Study phase	Status	Clinical Trials Reference
***Glucose metabolism inhibitors***					
Metformin	Nivolumab	III–IV NSCLC	II	Active, not recruiting	NCT03048500
	Nivolumab	Refractory MSS Colorectal Cancer	II	Active, not recruiting	NCT03800602
	Sintilimab	SCLC	II	Recruiting	NCT03994744
	Pembrolizumab	Advanced Melanoma	I	Recruiting	NCT03311308
	Nivolumab and	Solid Tumor	II	Recruiting	NCT04114136
	Pembrolizumab				
***Inhibitors of glutamine and glutamate pathway***					
Trigriluzole	Nivolumab and Pembrolizumab	Metastatic or Unresectable	I	Completed	NCT03229279
		Solid tumors or Lymphoma			
Telaglenastat	Pembrolizumab	KEAPl/NRF2-mutated,	II	Recruiting	NCT04265534
		stage IV, nonsquamous, NSLC			
	Nivolumab	Melanoma, ccRCC and NSCLC	I/II	Completed	NCT02771626
DRP-104	Atezolizumab	Advanced solid tumors	I/II	Recruiting	NCT04471415

Metformin (Various effects on glucose metabolism and levels); Trigriluzole (FC/BHV-4157; reduces extracellular glutamate levels by promoting uptake and inhibiting the release of this amino acid); Telaglenastat (CB-839, glutaminase 1 inhibitor); DRP-104 (Sirpiglenastat, a glutamine antagonist); Nivolumab (anti-PD-1 antibody); Sintilimab (anti-PD-1 antibody); Pembrolizumab (anti-PD-1 antibody); Pembrolizumab (anti-PD-1 antibody); Atezolizumab (anti-PD-L1 antibody); NSCLC, non-small-cell lung carcinoma; SCLC, small cell lung cancer; ccRCC, clear cell renal cell carcinoma.

### Targeting Glutamine Metabolism in Combination With PD-1/PD-L1 Checkpoint Blockade Immunotherapy for Tumor Treatment

Glutamine metabolism meets the metabolic needs of rapidly proliferating tumor cells. Similar to the glucose competition in the TME, there is also glutamine competition between tumor and immune cells, and glutamine metabolism directly affects tumor immune escape. A large number of basic and clinical experiments have shown that targeting of tumor glutamine metabolism can effectively inhibit tumor growth. Current strategies targeting glutamine metabolism mainly focus on key enzymes of glutamine metabolism, such as glutaminase (GLS), the glutamine transporter SLC1A5 and glutamate dehydrogenase 1 (GLUD1) (87–89). The relationship between tumor glutamine metabolism and immune escape provides a theoretical basis for the targeting of glutamine metabolism in combination with PD-1/PD-L1 immune checkpoint blockers for tumor treatment. Therefore, targeted of glutamine metabolism in combination with anti-PD-1/PD-L1 for tumor treatment will further improve the therapeutic effect, which is mainly based on the following ideas. ① The targeting of glutamine metabolism to inhibit the use of glutamine by tumors relieves the tumor’s restriction on the use of glutamine by T cells in the TME, which enhances the function of T cells. Combining with PD-1/PD-L1 checkpoint blockade immunotherapy may further improve the antitumor immune response. ② The targeting of glutamine metabolism directly affects the immune escape of tumors *via* different mechanisms, such as regulation of the generation of negative immune cells, improving the efficacy of anti-PD-L1 therapy and direct upregulation of the function of T_eff_ cells in TME. Therefore, combinations with PD-1/PD-L1 immune checkpoint inhibitor may further improve the antitumor effect. ③ Glutamine metabolism directly regulates PD-L1 expression in tumor cells and inhibits the function of T_eff_ cells. Combinations with anti-PD-L1 monoclonal antibodies can relieve this effect and improve the body’s antitumor immune response **(****Figure 3****)**.

Several compounds targeting glutamine metabolism have been developed for antitumor therapy. The glutamine antagonist DON and its precursor JHU-083 inhibit tumor cell proliferation *via* inhibition of the activity of a variety of enzymes that are required for tumor glutamine metabolism. JHU-083 has been proven to delay tumor growth and promotes the production of durable and highly active antitumor T cells. The combination of JHU-083 with PD-1 immune checkpoint inhibitors enhanced the antitumor effects (70). These results indicate the potential of targeting glutamine metabolism in combination with PD-1/PD-L1 checkpoint blockade immunotherapy for tumor treatment. Although no such combination therapy is available for clinical treatment, some related combination therapies are in clinical trials, including the combination of the GLS inhibitor CB-839 and a PD-1 ICI for cancer treatment (**Table 1**). CB-839 treatment also enhanced CTL-mediated antitumor responses in mouse models (90), which may explain the synergistic effect between CB-839 and PD-1 inhibitors. Renal cancer has been proven to constitute a glutamine-dependent tumor (91). The targeting of glutamine may be a potential treatment for renal cancer. However, one study found that deprivation of glutamine in the culture medium upregulate the expression of PD-L1 in renal cancers (61). In addition, it has been proved that targeting of glutamine metabolism can upregulate the expression of PD-L1 in lung cancer and colon cancer (62). Therefore, the targeting of glutamine metabolism in combination with PD-L1-targeted ICIs may produce a more powerful therapeutic effect in the future. Although the targeting of glutamine metabolism in combination with PD-1/PD-L1 immune checkpoint therapy showed promising results, similar to strategies targeting glucose metabolism, it is necessary to determine how to achieve maximum killing of cancer cells while minimizing the negative impact on normal and immune cells. Different drugs that target glutamine metabolism may have different effects on immune cells in TME. For example, JHU-083 and V-9302 inhibit glutamine metabolism in T_eff_ cells, but T cells regulate their own metabolism *via* different mechanisms without affecting their antitumor function (69, 70). Therefore, in addition to the combination of imaging technology and isotope labeling technology to solve the above mentioned problems, the development of more reasonable drugs that target glutamine metabolism without affecting or enhancing the function of T_eff_ cells on the basis of the difference in glutamine metabolism between tumor and T_eff_ cells is the focus of future research.

## Conclusion

Immunotherapy, especially PD-1/PD-L1 checkpoint blockade immunotherapy, have ushered in a new era of cancer treatment. Although immunotherapy is unique in its ability to achieve long-term and complete responses, most patients do not benefit from treatment. Therefore, it is necessary to examine new treatment strategies to further improve immunotherapy efficacy, such as the combination of immunotherapy with targeted therapy (92). The in-depth study of tumor and immune cell glucose and glutamine metabolism produced increasing evidence indicating that the metabolic interaction between the tumor cells and immune cells may be related to a poor response to immunotherapy. Therefore, the targeting of tumor glucose or glutamine metabolism in combination with PD-1/PD-L1 ICIs may provide new treatment opportunities for patients with tumors. However, we need to pay more attention to which types of tumors may benefit from combination therapy. Siska and colleagues classified tumors into four metabolic types using the metabolic-tumor-stroma score. Targeting glycolysis might be essential to allow an effective immune response in a mixed tumor type with glycolysis and OXPHIS (MeTS3) and a highly glycolytic Warburg type (MeTS4), which may benefit from combination therapy (19). There is no reliable method to judge glutamine metabolism; thus, more in-depth research is needed.

In this review, we consider the combination of targeted glucose or glutamine metabolic therapy and PD-1/PD-L1 checkpoint blockade immunotherapy for the treatment of tumors. These strategies are mainly based on the mutual regulatory relationship between the metabolism of cancer and immune cells in the TME. We discussed the metabolic competition between tumor and immune cells in the TME, the differences in metabolic reprogramming between these cells, the different effects of metabolism on tumor, T_eff_ and T_reg_ cells, and the effect of targeting tumor glucose or glutamine metabolism in the TME on the tumor immune response. In future studies, the relationship between metabolic reprogramming and tumor immune escape should be fully considered to optimize therapy and avoid problems, such as off-target immunity, drug resistance, and possible metastasis, and the ineffectiveness of targeted metabolic therapy. The targeting of glucose metabolism and glutamine metabolism may directly regulate the expression of PD-L1 in tumor cells (36, 61), which modulate the effects of targeted metabolic therapy because it may cause tumor immune escape. Therefore, the combined use of targeted glucose or glutamine metabolic therapy and PD-L1 ICIs may induce a synergistic effect. However, the interaction between metabolism and tumor immune escape has additional effects. The intricate relationship between metabolism and immune escape reflects the difficulty of targeting the metabolic adaptations of tumor cells without affecting tumor clearance by T_eff_ cells. Therefore, it is necessary to fully examine the metabolic mechanism of tumor immune escape, the metabolic requirements of immune cells, and the relationship between PD-1/PD-L1 immune checkpoints and metabolism to evaluate the impact of targeted glucose or glutamine metabolic therapy on immune checkpoints and the impact of immune checkpoint treatment on tumor metabolism. Designing a more reasonable combination treatment plan based on the metabolic crosstalk between tumor and immune cells can avoid treatment failure and increase the effectiveness of PD-1/PD-L1 checkpoint blockade immunotherapy in more cancers.

## Author Contributions

GM, CL, HN, and WS collected articles and designed the manuscript. GM, CL, and ZZ wrote the manuscript. YL and ZL prepared tables and figures. YC, LW, DL, and MZ revised and approved the manuscript. All authors contributed to the article and approved the submitted version.

## Funding

This study was financially supported by the National Natural Science Foundation of China (82071750, 81772713, 81472411), Taishan Scholar Program of Shandong Province (tsqn20161077), Major Science and technology innovation project of Shandong Province (2019JZZY021002), Key projects of Qingdao Science and Technology Program (18-6-1-64-nsh), Research and Development Program of Shandong Province (2018GSF118197).

## Conflict of Interest

The authors declare that the research was conducted in the absence of any commercial or financial relationships that could be construed as a potential conflict of interest.
